# Cardiovascular effects of H_3_ histamine receptor inverse agonist/ H_4_ histamine receptor agonist, clobenpropit, in hemorrhage-shocked rats

**DOI:** 10.1371/journal.pone.0201519

**Published:** 2018-08-02

**Authors:** Bartosz Wanot, Karolina Jasikowska, Ewa Niewiadomska, Agnieszka Biskupek-Wanot

**Affiliations:** 1 Polonia University, Health and Nursing Institute, Częstochowa, Poland; 2 Department of Physiology, School of Medicine with the Division of Dentistry in Zabrze, Medical University of Silesia in Katowice, Zabrze, Poland; 3 Department of Biostatics, School of Public Health in Bytom, Medical University of Silesia in Katowice, Bytom, Poland; 4 Specialist Dental Clinic, Czestochowa, Poland; Max Delbruck Centrum fur Molekulare Medizin Berlin Buch, GERMANY

## Abstract

Hemorrhagic shock has a potential to be life-threatening when it is not treated. The main causes of hemorrhagic shock involve: (1) forces causing injury; and (2) diseases that can cause hemorrhage., Therefore, due to the causes of hemorrhagic shock and the life-threatening potential, the search for new methods of shock treatment is extremely valuable to the modern medicine. The aim of this study was to investigate the influence of clobenpropit in the model of hemorrhagic shock. The experiments were conducted in 110 adult male Wistar rats weighing between 205 and 470g. 1, 2 and 5 μmol/kg of intravenous H_3_ receptors reverse agonists, clobentropit, and/or 1, 5 and 10 μmol/kg H_3_ receptor agonist, imetit, were used as general anesthetics. Irreversible hemorrhagic shock was induced by the paused bleeding until the mean arterial pressure (MAP) lowered to the level of 20–25 mmHg. It was proved that, in cases of critical hypotension, clobenpropit triggered a dose-dependent increase of: systolic blood pressure (SBP), diastolic blood pressure (DBP), MPA and heart rate (HR) of rats with critical hypotension. The most significant changes in hemodynamic parameters were achieved by administrating dosages of 2 mmol/kg. This resulted in the survival rate increase to up to 100%. However, imetit did not trigger any hemodynamic changes nor an increase in SBP, DBP, MAP or HR. Furthermore, it was found that the premedication with prazosin, yohimbine, 6-hydroxydopamine and the vasopressin V_1a_ receptor antagonist blocked the effects of clobenpropit. Additionally, premedication with propranolol, captopril and ZD 7155 did not cause any significant changes in the measured hemodynamic parameters. In conclusion, after an intravenous injection clobenpropit, the inverse agonist of H_3_ histamine receptors/agonist of histamine receptors H_4_, causes a resuscitating effect on rats in hemorrhagic shock. Moreover, such effect is based on the effector mechanisms of sympathetic nervous system and vasopressin.

## Introduction

Histamine is a heterocyclic amine deriving from imidazole. It can be found in almost all mammal tissues, however high concentration of histamine is discovered in the peculiar localizations including skin, intestines and mucosa of the bronchi. In the human body histamine is mainly stored in mast cells and basophils. It can additionally be found in histaminergic neurons in the central and peripheral nervous system. Histamine operates through the activation of specific membrane receptors. Currently, to the present date, four types of histamine receptors are known and they include H_1_, H_2_, H_3_ and H_4_ histamine receptors.

It has been proved that clobenpropit suppresses perivascular nerves’ stimulation caused by the selective agonist of H_3_ receptor − α-methylhistamine. This results in the increase of the noradrenaline secretion from the endings of the sympathetic nerves [1] and the inhibition of mesenteric arteries vasorelaxation in rats [2]. Furthermore, it was demonstrated that clobenpropit causes increased secretion of noradrenaline from the postganglionic endings of the sympathetic nervous system, as well as the occurrence of cardiac arrhythmias in guinea pigs [3].

Shock is a life-threatening condition, characterized by abnormal perfusion on the microcirculation level, which is a result of disproportion between the metabolic demand of tissues and blood flow within tissues. It leads to an insufficient cell nutrition, oxygen deprivation and inadequate removal of metabolic products [4]. In the first phase of haemorrhagic shock, the body utilises own compensatory mechanisms to minimise the results of shock. In this phase, the sympathetic nervous system is stimulated by baroreceptors. As a result, the maintenance of arterial blood pressure is achievable. In the second phase, the sympathetic nervous system's suppressive apparatus becomes active. This is rapidly followed by the expansion of capillaries and decrease in arterial blood pressure [5].

Considering the above post-haemorrhagic risks in humans, including death, it must be stressed here that further research is of the highest importance in search for treatment options. It can be suggested, that this further research examines pathophysiologic mechanisms that are yet to be discovered.

The presented research focuses on the importance of H_3_ histamine receptors, in particular, on the role of its inverse agonist, clobenpropit, in cardiovascular compensation examined in the experimental model of hemorrhagic shock.

## Material and methods

The experiment protocol was accepted by the Local Ethic Committee for Animal Experiments in Katowice (Notification No. 39/2009, 30/2012 and 31/2012). All surgery was performed under ketamine and xylazine anesthesia, and all efforts were made to minimize the suffering of the animals. Research was financed by the 2011 PhD funds, number KNW-1-042/D/1/0.

### Laboratory animals

The examination was conducted in 110 adult male Wistar rats, weighing 205–470 grams. Rats were provided by the Centre of Experimental Medicine of the Medical University of Silesia in Katowice. Until the beginning of the examination, animals were kept in separate cages in the animal quarters. Fixed temperature of 20–22°C and 12-hour day/night cycle with food and water *ad libitum* were provided.

### Preparatory procedures

All experiments on the animals were carried out under general anesthesia induced with ketamine (100 mg/kg) and xylazine (10 mg/kg), and administered intraperitoneally. A generally accepted procedure was used to cannulate the lateral ventricle of the brain.10 μl 0,5% of methylene blue was administered to verify the correct location of canulae. Animals with the absent methylene blue brain penetration were not included in the study.

Cannulas filled with heparinized solution of 0.9% NaCl (100 IU/ml) were inserted into the dissected right carotid artery and jugular vein. This allowed the application of the direct method of blood pressure measurement and the induction of bleeding resulting in hemorrhagic shock. The animals were monitored for the total of two hours.

### Measurement of cardiovascular system parameters

SBP, DBP, MAP and HR were measured with blood pressure gauge Transducer Amplifier Module TAM-A (Hugo Sachs Electronic, March-Hugstetten, Germany).

After performing laparotomy, renal blood flow (RBF) was measured. This was achieved by dissection of the right renal artery In addition, electromagnetic sensor (type 1 RB, Hugo Sachs Electronic, March-Hugstetten, Germany) connected with the measuring set of Transit Time Flowmeter Type 700 (Hugo Sachs Electronic, March-Hugstetten, Germany) was placed.

To measure skeletal muscle microcirculatory flow (SMMF) the skin and the subcutaneous tissue were incised. A needle electrode was inserted into the quadriceps of the back right limb. The flow was measured using a microflow gauge (Perfusion System, Oxford Optronix Ltd., Oxford, Great Britain). All parameters were measured following a 30min period of adaptation.

### Hemorrhagic shock model

An irreversible hemorrhagic shock was induced in accordance with the Guarini’s et al. modified method [6,7]. This method involved 15–25 minutes of intermittent bleeding through the cannula in the right jugular vein. Polythene drain was used and the MAP was monitored until it reached the reduced and stable level of 20–25 mmHg. The subject was bled approximately every 3–5 minutes for 1 to 2 minutes at a time and achieving a maximum blood flow rate of 1 ml/min.

### Humane endpoints

During the entire study (lasting 2 hours) the animals were anesthetized with ketamine and xylazine. The hemodynamic parameters of the animals were monitored throughout the study period (SBP, DBP, MBP and HR). Death of an animal was a planned experimental endpoint. At the end of the experiment, all animals in deep anesthesia were euthanized by exsanguination through the previously cannulated right carotid artery [5,6]. During the experiment, all animal welfare considerations were undertaken, including efforts to minimize suffering and distress. Local Ethic Committee for Animal Experiments in Katowice approved the anticipated mortality in the study design.

### Examination protocol

In normotensive rats, Clobenpropit was administered in doses of 1, 2 and 5 μmol/kg intravenously; otherwise, it was administered in the 5^th^ minute of the critical hemorrhagic hypotension [8]. This allowed the comparison of hemodynamic effects resulting from the blockage of H_3_ histamine receptors in normotension and hemorrhagic hypotension.

To examine the influence of the chemical sympathectomy on the pressor effect caused by clobenpropit, in normotensive rats there was subcutaneously administered a 6-hydroxydopamine (50 mg/kg (SC) for 3 consecutive days. In the fourth day following the shock 2 μmol/kg (IV) of clobenpropit was administered [9].

To examine the action of the (1) sympathetic nervous system, (2) the renin-angiotensin system and (3) vasopressin in the pressor effect caused by clobenpropit, immediately after the end of the bleeding, separate groups of receptor antagonists were administrated. Those included: (1) α_1_-adrenergic–prazosin (0.5 mg/kg, iv), α_2_-adrenergic–yohimbine (1 mg/kg, iv) and β-adrenergic–propranolol (1 mg/kg, iv), (2) angiotensin I type–ZD 7155, and the enzyme inhibitor of converting angiotensin–captopril (30 iv mg/kg), and (3) vasopressin V_1a_ type–[β-mercapto-β, β-cyclopentanemethylpropionyl-O-methyl-Tyr, Arg] AVP (10 μg/kg; iv), and 5 minutes later–clobenpropit (2 μmol/kg, iv) [5,10, 11].

Doses of the applied tooling compounds were selected on the basis of the literary activity [5, 8–12].

The following parameters were examined in all groups of animals: MAP, SBP, DBP, HR, RBF and SMMF.

In order to mark the epinephrine and norepinephrine concentrations in the blood plasma, samples of 0.6 ml of blood were taken from the right jugular vein. The blood was sampled at the beginning of the bleeding, post bleeding and 20 minutes post the intravenous administration of clobenpropit and the NaCl solution. Equal volume of the 0.9% NaCl solution was respectively administered to animals to replace the lost blood. The markings of catecholamines were carried out with the radioimmunoassay method on the automatic meter RIA LKB Gamma Counter. For the examinations, reagents of the LDN Company Germany were applied.

### Applied substances

The following substances were used in the experiments: hydrochloride yohimbine, hydrochloride (±)–propranolol (Research Biochemicals Inc., Natick, USA), hydrochloride ketamine, xylazine (Vétoquinol Biowet, Gorzów Wielkopolski), heparin (Polfa, Warsaw), [β-mercapto-β, β-cyclopentanemethylpropionyl-O-methyl-Tyr, Arg] AVP, captopril, hydrochloride prazosin, hydrobromide 6-hydroxydopamine (6-OHDA), double-hydrobromide, clobenpropit, ZD 7155 (Tocris Bioscience, Bristol, Great Britain).

All the compounds, with the exception of prazosin and 6-hydroxydopamine, were dissolved in the 0.9% NaCl solution; nonetheless, the prazosin was dissolved in double-distilled water; yet, 6-hydroxydopamine in the 0.9% NaCl solution containing 0.1% of ascorbic acid. All the solutions were prepared on the day of the examination.

### Statistical methods

For the presentation of quantitative variables, the arithmetic mean and the standard error of the mean (SEM) were described. The compatibility of the distribution of continuous variables with the normal distribution was verified with the Shapiro-Wilk compatibility test. For the evaluation of the intergroup differences relevance in the analyzed quantitative variables the statistical significance tests of differences were carried out. Parametric tests were applied to the normal distribution of the studied features: the t-Student test for a single sample, in the case of two groups—the t-Student test, whereas in the case of related variables the t-Student test for dependent variables. The analysis of many groups was conducted using the variance analysis (ANOVA) and post-hoc tests. The nonparametric test was applied in the case of diverged data from the normal distribution: the Mann-Whitney U test for two unrelated groups, the Wilcoxon test for dependent variables, whereas in the analysis of many unrelated groups—the Kruskal-Wallis tests as well as the multiple comparison tests. An analysis of the many related groups was carried out using the Friedman test. The direct test of Fisher was used to evaluate the differences relevance in survival rate. The level of statistical significance was determined by p < 0.05.

## Results

Average body weight of the rats was 307.8 ± 5.7 g. The initial mean values were: HR 246 ± 4.9 contraction/minute, SBP 128.1 ± 2.9 mmHg, DBP 85.5 ± 2 mmHg, MAP 101.6 ± 2.4 mmHg, RBF 3.5 ± 0.09 ml/minute and SMMF 170.8 ± 8.9 BPU (blood perfusion units). In order to achieve the hypotension of 20–25 mmHg, the average volume of the released blood was 2.44 ± 0.06 ml/ 100 g of the body weight.

### Influence of clobenpropit on hemodynamic parameters in normotension and critical hemorrhagic hypotension

**Fig 1:**
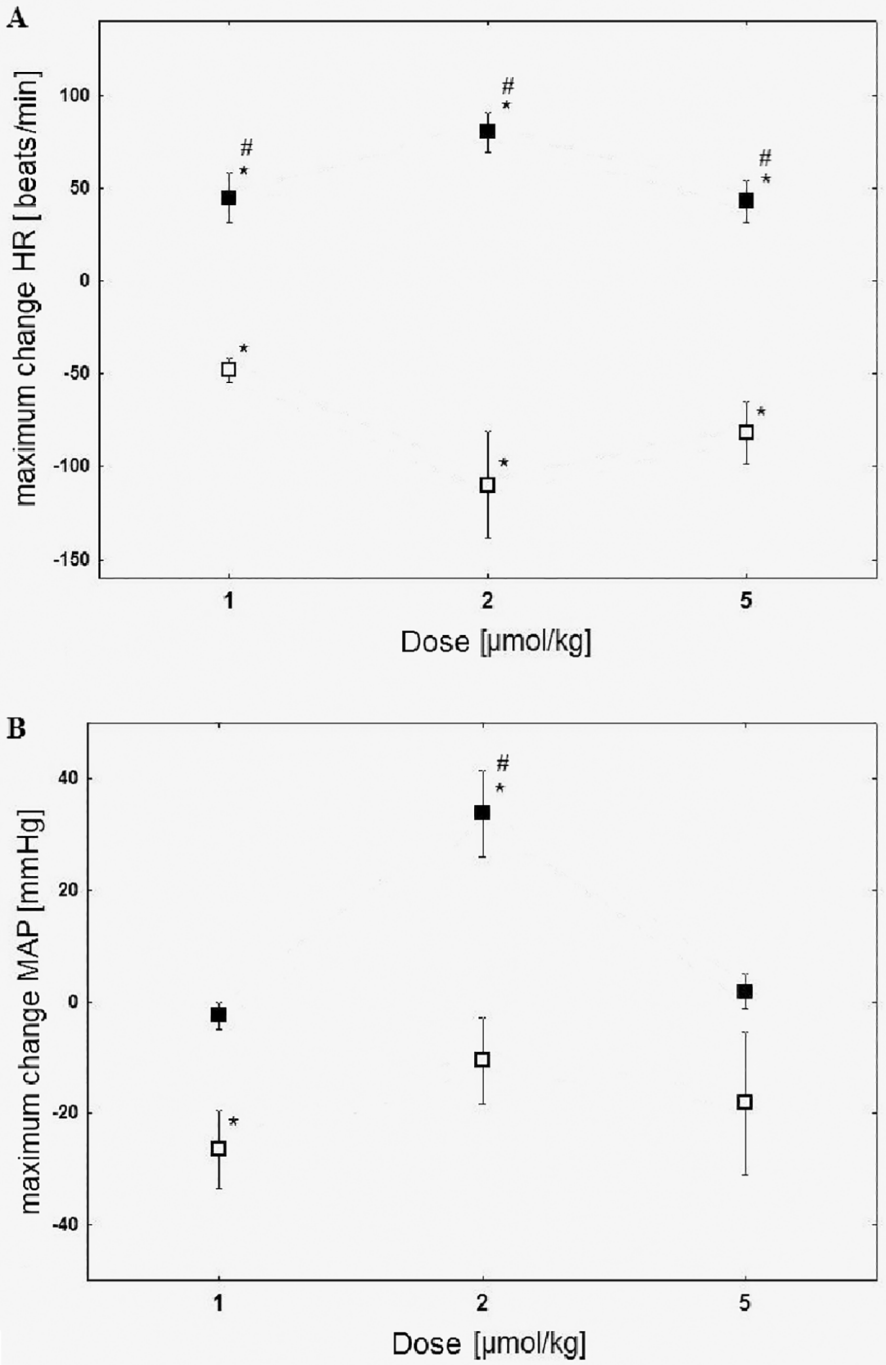
Maximum changes of heart rate (HR) (A) and mean arterial pressure (MAP) (B) post intravenous clobenpropit injection (1, 2 and 5 μmol/kg) in normotensive rats (□) and in critical hemorrhagic hypotension (■); n = 5; medium ± SEM;*p < 0.05 compared to initial values; #p < 0.05 compared to the normotensive animals group.

Fig 1 illustrates the maximum changes of HR and MAP after the intravenous clobenpropit administration in normotensive animals and in the critical hemorrhagic hypotension.

In the group of the normotensive animals clobenpropit administered at a dose of 1, 2 and 5 μmol/kg did not lead to any significant change nor caused a decrease in HR or MAP (Fig 1). In the group of animals with the critical hypotension, clobenpropit triggered a statistically significant increase of MAP (dose 2 μmol/kg) and HR (doses of 1, 2, 5 μmol/kg) (Fig 1). The biggest changes of the hemodynamic parameters were observed after the administration of a 2 μmol/kg dose. This was in conjunction with a 100% increase in rats’ survival rate within the critical hemorrhagic hypotension group.

### Influence of premedication using antagonists of α_1_- α_2_- and β-adrenergic receptors on the changes of hemodynamic parameters post clobenpropit administration

**Fig 2:**
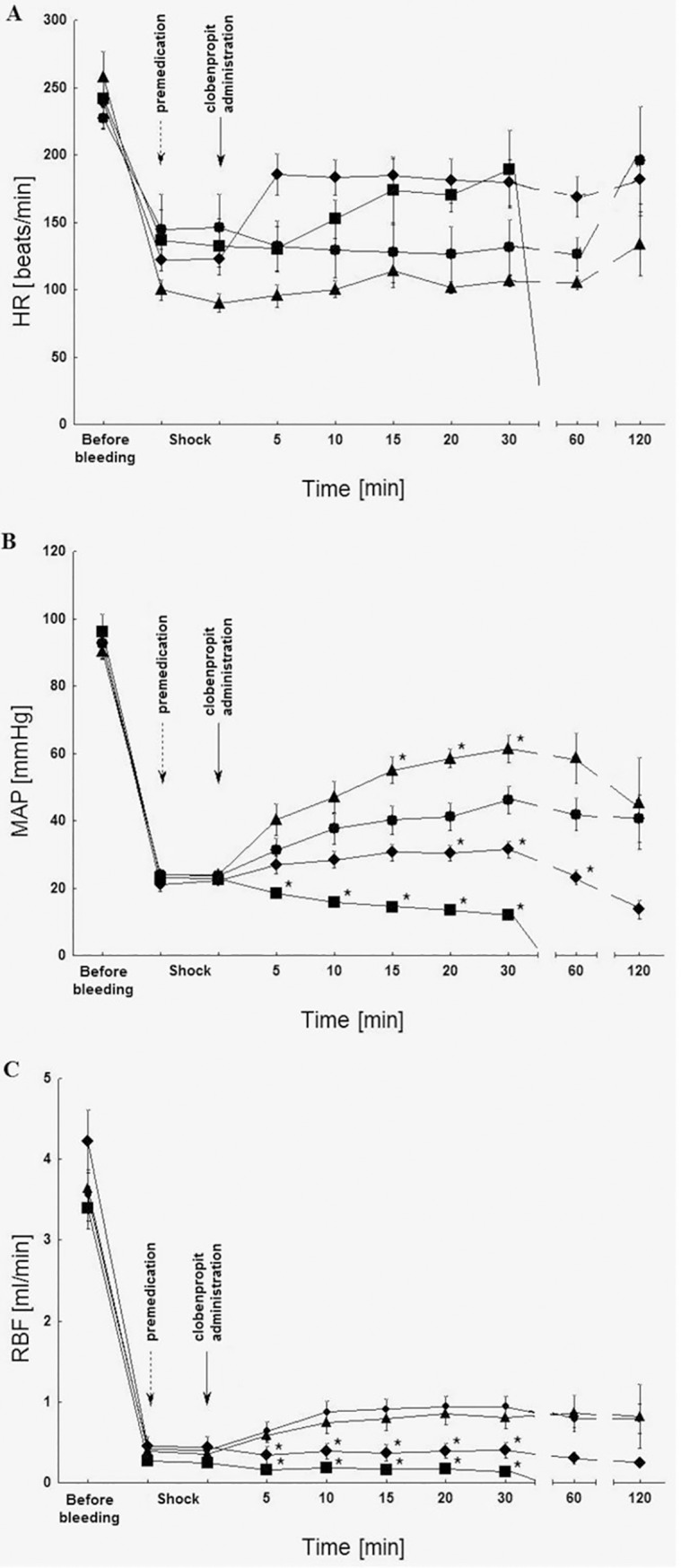
Influence of prazosin (0.5 mg/kg iv; ■), yohimbine (0.5 mg/kg iv; ♦), propranolol (1 mg/kg iv; ▲) premedication on the heart rate (HR) (A), mean arterial pressure (MAP) (B) and renal blood flow (RBF) (C) post clobenpropit administration (2 μmol/kg iv; ●) in rats in the critical hemorrhagic hypotension; n = 5; medium ± SEM.

Changes of hemodynamic parameters post clobenpropit administration and the premedication with prazosin, yohimbine and propranolol are illustrated on Fig 2.

It can be observed, that the premedication with the receptor antagonist of α_1_-adrenergic prazosin (0.5 mg/kg) and α_2_-adrenergic yohimbine (0.5 mg/kg) significantly reduced the changes of MAP and RBF induced by clobenpropit (2 μmol/kg) (Fig 2B and 2C). The influence of prazosin also caused a decrease in the survival rate of 2 hours to 0%; however, yohimbine did not affect the survival rate of 2 hours.

In comparison with the group in which clobenpropit alone was administered (2 μmol/kg), Propranolol (1mg/kg) temporarily increased the growth of MAP (Fig 2B). However, it did not affect HR (Fig 2A), RBF (Fig 2C) nor the survival rate of 2 hours.

### Influence of the chemical sympathectomy on the changes of hemodynamic parameters post clobenpropit administration

**Fig 3:**
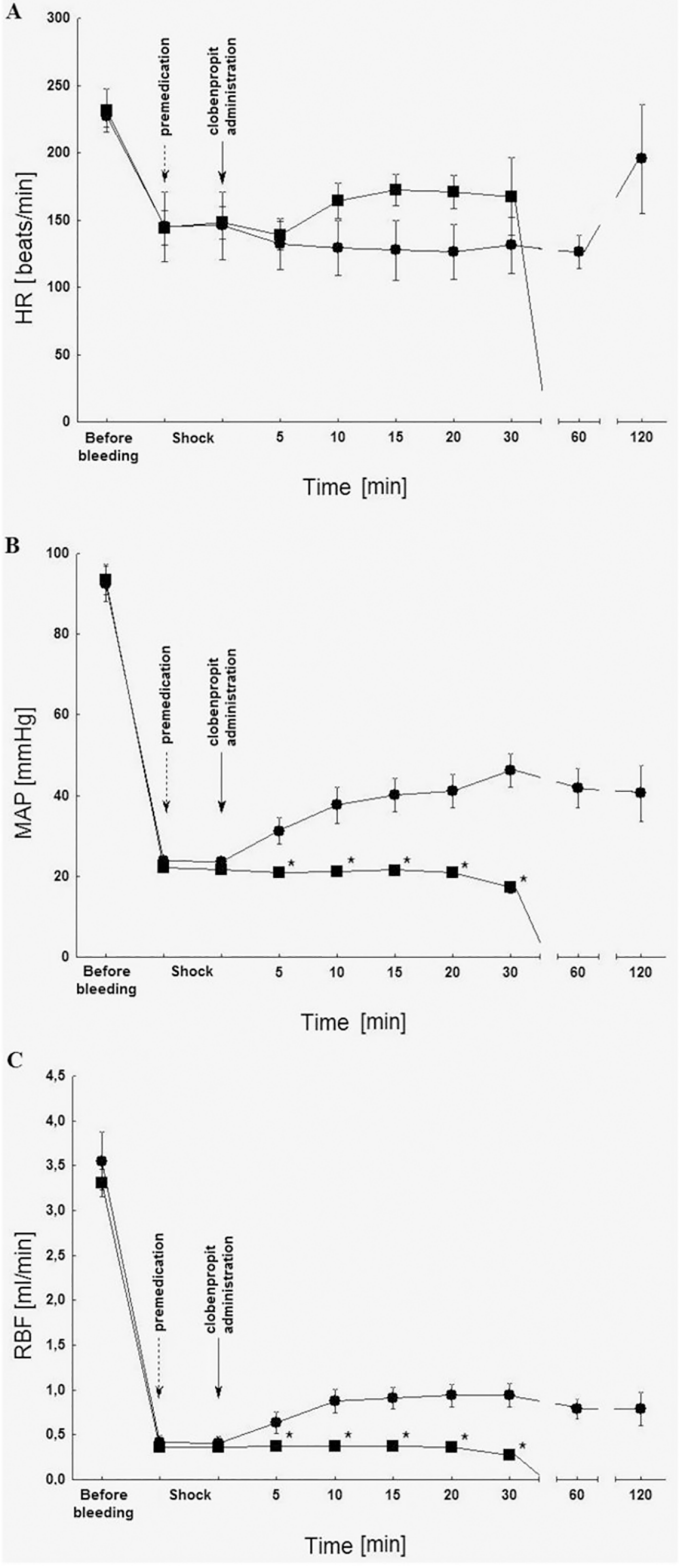
Influence of 6-hydroxydopamine premedication (50 mg/kg for 3 consecutive days; ■) on heart rate (HR) (A), mean arterial pressure (MAP) (B) and renal blood flow (RBF) (C) after clobenpropit administration (2 μmol/kg iv; ●) in rats in the critical hemorrhagic hypotension; n = 5; ± SEM.

Changes of hemodynamic parameters post clobenpropit administration and the 6-hydroxydopamine premedication can be found on Fig 3.

The 6-hydroxydopamine premedication (50 mg/kg for 3 consecutive days), which caused chemical sympathectomy, significantly inhibited the increase of MAP. Furthermore, it repressed the RBF induced by clobenpropit (2 μmol/kg) (Fig 3B and 3C). Finally, the use of 6-hydroxydopamine led to a decrease in the survival rate to 0%.

### Influence of the premedication with angiotensin receptor antagonist type I and with the inhibitor of the angiotensin-converting enzyme, on the changes of hemodynamic parameters post clobenpropit administration

The premedication with the antagonist of the angiotensin type I ZD 7155 (0.5 mg/kg) receptor, as well as captopril (30 mg/kg), did not demonstrate statistically significant changes in the measured hemodynamic parameters in relation to the group, in which clobenpropit alone was administered (2 μmol/kg 1). It did not affect the survival rate of 2 hours, either.

### Influence of the premedication with antagonist of vasopressin type V_1a_ receptor on the changes of hemodynamic parameters after clobenpropit administration

**Fig 4:**
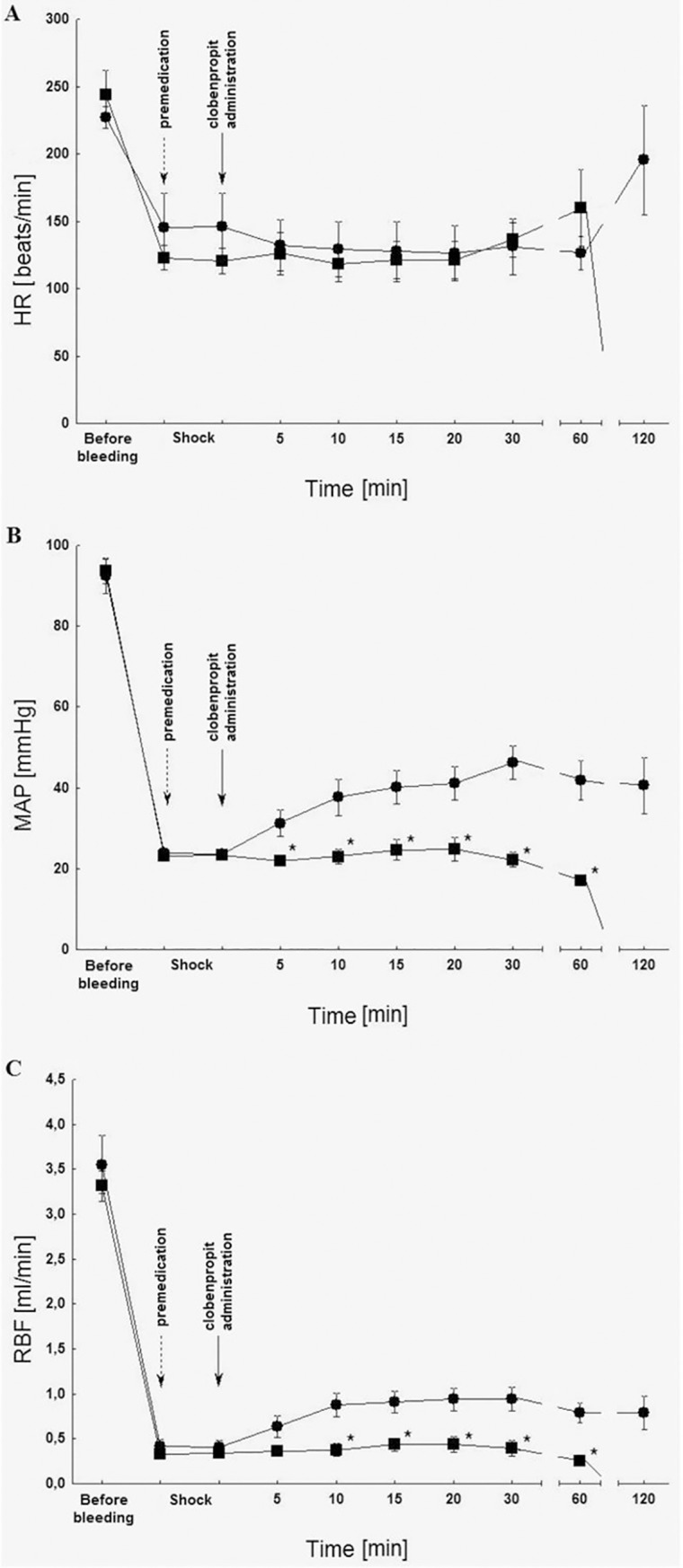
Premedication influence [β-mercapto-β, β-cyclopentanemethylpropionyl-O-methyl-Tyr, Arg] AVP (10 μg/kg iv; ■) on heart rate (HR) (A), mean arterial pressure (MAP) (B) and renal blood flow (RBF) (C) post clobenpropit administration (2 μmol/kg iv; ●) in rats in the critical hemorrhagic hypotension; n = 5; medium ± SEM.

On Fig 4 the changes of hemodynamic parameters post clobenpropit administration and the premedication of [β-mercapto-β, β-cyclopentanemethylpropionyl, O-methyl-Tyr, Arg] AVP were described.

Premedication with the antagonist of vasopressin V_1a_ type receptor (10 μg/kg) significantly inhibited the increase of MAP and RBF in relation to the group, in which clobenpropit alone was administered (2 μmol/kg) (Fig 4B and 4C). Premedication with antagonist of vasopressin type V_1a_ receptor also led to a decrease in the survival rate to 0%.

### Concentrations of catecholamines in blood plasma

Statistically significant changes in adrenaline concentrations have been shown 20 minutes post administration of clobenpropit and NaCl. No significant variances have been noted prior or post the induction of shock.

Furthermore, no substantial changes between the concentrations of norepinephrine in the examined time points were observed.

According to the recommendations of the Local Ethical Committee of Animal Experiments Department in Katowice, animal examinations in control groups were not conducted. Reference to the previously published results has been applied.

## Discussion

In normotensive animals clobenpropit either caused a reduction of MAP or did not affect this parameter, which was consistent with the results of the previous studies of Obuchowicz et al. [8]. In contrast to these results, clobenpropit administered in hemorrhagic hypotension in a dose of 2 μmol/kg resulted in a long-lasting increase of blood pressure, as well as the increased survival rate after 2 hours up to 100%. This suggests a new discovery, which indicates a resuscitation potential of the H_3_ receptor inverse agonists in the hemorrhagic shock model in rats.

As previous studies showed, IV administration of clobenpropit presents the ability to penetrate through the blood-brain barrier [13]. In order to verify whether the observed pressor effect is of the central or peripheral origin, clobenpropit was administered to the lateral ventricle of the brain. It was shown that after the central administration of clobenpropit, no significant effect on the measured hemodynamic parameters occurred in animals post hemorrhagic shock. In contrast, studies conducted by Chen [14] showed that clobenpropit, administered to the lateral ventricle of the brain, causes an increase in the regional cerebral blood flow in rat’s hippocampus [14].

In order to identify the effector systems involved in the clobenpropit response, the pharmacological methods were applied (the peripheral administration of tool substances–antagonists of selected receptors) to demonstrate the stimulation of the sympathetic nervous system, the renin-angiotensin system, as well as an increase in the vasopressin secretion post administration of the H_3_ receptor inverse agonist.

H_3_ histamine receptors within the peripheral nervous system are located in a large number in postganglionic fiber endings of the sympathetic nervous system, which innervate the heart and blood vessels. Appearing locally, histamine demonstrates an effect on the secretion of noradrenaline in the sympathetic nervous system. This system has a primary importance among the compensation mechanisms activated during the resuscitating effect, caused by the centrally-acting melanocortin peptides [15], histamine [16] and leptin [17]. However, unlike the action of the afore-mentioned neuromodulators/neurotransmitters this research demonstrated a potential of activation of the sympathetic nervous system resulting from the peripheral clobenpropit effect. It was stated that the use of the selective antagonist of α_1_-adrenergic receptor, prazosin, entirely blocks the pressor reaction caused by clobenpropit. This manifests the importance of α_1_-adrenergic receptors, as a basic component in the pathophysiology chain reaction, which was responsible for the observed resuscitating effect. It was found that blocking α_2_-adrenergic receptors with yohimbine limits further the pressor reaction of clobenpropit, however, it does not affect the 2 hours survival rate. This may suggest the participation of the α_2_-adrenergic receptors in the pressor effect of clobenpropit. Their role in the shock was repeatedly proven, since it was presented that those receptors participate in activating the blood staying in containers of venous part of the cardiovascular system, i.e. in the liver, spleen and large veins. Such action was shown previously in relation to the histamine [16], ACTH [15] and leptin effects [17] in hemorrhagic shock.

As opposed to antagonists of α_1_- and α_2_-adrenergic receptors, propranolol was not found to block the resuscitating effect of clobenpropit. These results are consistent with the earlier examinations, in which it was shown that changes in heart rhythm do not play a significant role in the resuscitating mechanisms of shock; but however, changes of the peripheral vascular resistance and circulating blood volume have a primary importance.

The conducted biochemical examinations (marking of adrenaline and noradrenaline concentration in blood plasma) did not demonstrate statistically significant changes in the concentration of the examined catecholamines pre- and post bleeding induction. The lack of differences among groups in the levels of concentration of catecholamines in blood pre-hemorrhage is an expected result. Additionally, it confirms the homogeneity of the examined rats’ population. However, the absence of statistically significant adrenaline and noradrenaline concentrations after hemorrhage was unanticipated. In contrast to this study, Jochem [17] confirmed that along with decrease in blood pressure as a result of hemorrhage, increase in adrenaline and noradrenaline concentration occurs [16]. Despite the fact that in the current research an increase in concentration of both catecholamines was demonstrated after the shock induction, due to (1) a small number of the examined groups (n = 5); and (2) great scattering of results, and thus, a standard error of the mean; this study failed to show statistical significance (adrenaline concentration: 37.95 ± 9.6 vs. 91.78 ± 32.8; noradrenaline concentration 52.94 ± 11.5 vs. 60.67 ± 10.4). It is worth noting that the noradrenaline concentrations in the control group and in the group, in which clobenpropit was administered in the twentieth minute of the observation, do not differ. Although such a result in the control group proves the repressive activity of the sympathetic nervous system this may be suggested as resulting from catabolism of endogenic noradrenaline secreted under the influence of clobenpropit.

It may perhaps be observed without straying too far afield from the primary focus that differences in adrenaline concentrations were noticed in the twentieth minute of the observation. It was demonstrated that in the control group this concentration was significantly higher than in the examined group. This can be explained by the fact that the animals in the control group remained continuously in a state of critical hypotension, whereas in the examined group MAP was about 40 mmHg, i.e. about twice as high as in the control group. Therefore, it may be suggested that the basic stimulus that increases the adrenaline secretion (the decompression of baroreceptors) was smaller in the examined group. It is necessary to emphasize that it was repeatedly proved that there is no electric activity repression in preganglionic fibers of the sympathetic nervous system innervating the adrenal medulla in the blockade phase of the sympathetic nervous system’s activity the hemorrhagic shock [16]. Hence, a high adrenaline concentration in the blood plasma is characterized by the blockade phase of the sympathetic nervous system in hemorrhagic shock.

It was proved in this study that carrying out the chemical sympathectomy by applying 6-hydroxydopamine entirely obstructs the presser reaction caused by clobenpropit. The application of chemical sympathectomy is used as a standard procedure in research concerning the sympathetic system’s activity. Its usefulness was demonstrated in inducing Parkinson's disease model [18], examining the role of dopamine in encephalon [19] and in the opioids’ activity [20]. The mechanism of administered measures to induce the sympathectomy to block of the production of catecholamines, affects their neuronal transport or the secretion from synaptic endings. It may be assumed that 6-hydroxydopamine causes not only sympathectomy, but also damage to dopaminergic and noradrenergic neurons by producing reactive forms of oxygen. This results in oxidative stress by the suppression of the complex I and IV of the respiratory chain in mitochondrions. According to the previous theories, as suggested by Kostrzew and Jacobowitz [21], 6-hydroxydopamine cannot only be stored in intracellular granularities, it may however produce reactive oxygen forms in the cytoplasm, Furthermore, those oxygen forms have a potential to damage intracellular structures,. This study demonstrated that 6-hydroxydopamine blocks the presser reaction caused by the H_3_ receptor inverse agonist, which may indicate that the norepinephrine secretion from the sympathetic endings plays a vital role in inducing the resuscitating effect in hemorrhagic shock.

A further effector mechanism in the hemorrhagic shock, which was analyzed, is the renin-angiotensin system. It has been widely accepted that this system operates at the systemic level, as well as locally–on the level of the tissues. In the case of hemorrhagic shock it must be assumed that it is the systemic renin-angiotensin system that is an effector mechanism, which can be activated. The factors increasing the renin secretion from the cells of the juxtaglomerular apparatus of kidneys include the stimulation of baroreceptors within granular cells (as a result of the reduced tension in the arterioles bondage post the perfusion pressure drop), the stimulation of chemoreceptors of the specks dense (as a result of the reduction in sodium and chloride ion concentration in the concentrated primitive urine in the shoulder stopping by loop of Henle), the local prostaglandins’ effect (especially prostacyclin − PGI_2_) and nitric oxide (NO) produced in the kidney cortex. In addition, factor increasing the renin secretion is the stimulation of receptors β_1_-adrenergic in granule cells, being a result of the systemic sympathetic stimulation and secreted locally noradrenaline Appreciably, the blood loss led to a hypotension, and thus to an increase of the renin secretion resulting from of the stimulation of baroreceptors of the juxtaglomerular apparatus. Fundamentally, this may lead to an increase in PGI_2_ and nil secretion as a result of the kidney cortex ischemia. Consecutively, clobenpropit can directly increase the noradrenaline secretion, which will stimulate β_1_-adrenergic receptors and thus increase the renin secretion. However, the current research on the renin-angiotensin system’s contribution showed the lack of the effect on the receptor antagonist administration of angiotensin type I and ZD 7155. Moreover, it was demonstrated that the inhibitor of the enzyme converting angiotensin captopril on the resuscitation of clobenpropit was not affected by that receptor. This deficiency can be a consequence of limited duration of clobenpropit exposure and by an insufficient amount of the secreted noradrenaline within the kidneys. In contrast to the current results, the previous studies proved that in the case of central histamine in hemorrhagic shock the blocking of the angiotensin-converting enzyme and angiotensin receptors demonstrates a blocking activity on the induced resuscitating effect [20]. One can unmistakably comprehend this difference having awareness of the mechanisms of histamine administration to the lateral ventricle of the brain. It demonstrates a very strong central stimulation of the sympathetic nervous system, which within a few minutes induces a total reversibility of the critical hemorrhagic hypotension [7].

Finally, the last analyzed effector mechanism is vasopressin. Vasopressin has a meaningful effect on clobenpropit. This hormone is produced in the supraoptic nuclei and, in smaller quantities, in the paraventricular nucleus of hypothalamus, from where it is transferred by axonal transport to the rear of the pituitary gland, where it is released. factors increasing the vasopressin secretion which must be taken into consideration in the case of hypovolemia entail: lowering of blood pressure and reducing the filling of the auricles with blood during their diastole (the reduced stimulation of volume-receptors auricles).

In the recent years, experimental and clinical studies have been carried out, in which the resuscitating properties of administered intravenously vasopressin and its analogues in hemorrhagic shock were confirmed [10]. This study found that the premedication with the use of the receptor antagonist of vasopressin V_1a_ type leads to the reduction of an amplitude of the presser reaction caused by clobenpropit. This may indicate that present vasopressin model has also an effector role. However, at the current stage of examination, the mechanism of the observed phenomenon remains unidentified. It can however be suggested, that the central effect of clobenpropit on H_3_ receptors acting as heteroreceptors may induce an increase in the vasopressin secretion. Similar effects were observed earlier in the reference to the central histamine in hemorrhagic shock in rats.

In conclusion, after an intravenous administration, clobenpropit caused a resuscitating effect in rats subjected to the hemorrhagic shock. The effector mechanisms, involved in the resuscitating effect of clobenpropit, involve both, the sympathetic nervous system and vasopressin.

## Supporting information

S1 File NC3Rs ARRIVE guidelines checklist 2014. (DOCX)

S2 File Plos-one-humane-endpoints-checklist. (DOCX)
