# Supplementary material for: Cardiovascular effects of H3 histamine receptor inverse agonist/ H4 histamine receptor agonist, clobenpropit, in hemorrhage-shocked rats
Source: PLoS One. 2018 Aug 2;13(8):e0201519. doi: 10.1371/journal.pone.0201519 (PMC6072086; doi:10.1371/journal.pone.0201519)

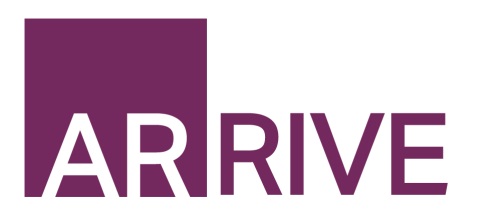


The ARRIVE Guidelines Checklist

Animal Research: Reporting In Vivo Experiments

Carol Kilkenny^1^, William J Browne^2^, Innes C Cuthill^3^, Michael Emerson^4^ and Douglas G Altman^5^

*^1^The National Centre for the Replacement, Refinement and Reduction of Animals in Research, London, UK, ^2^School of Veterinary Science, University of Bristol, Bristol, UK, ^3^School of Biological Sciences, University of Bristol, Bristol, UK, ^4^National Heart and Lung Institute, Imperial College London, UK, ^5^Centre for Statistics in Medicine, University of Oxford, Oxford, UK.*

|  | | ITEM | RECOMMENDATION | Section/ Paragraph |
| --- | --- | --- | --- | --- |
| 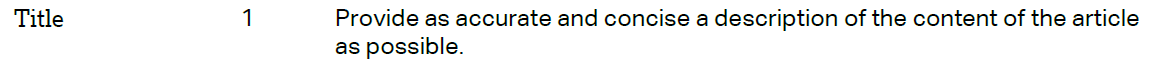 | | | Title |  |
| 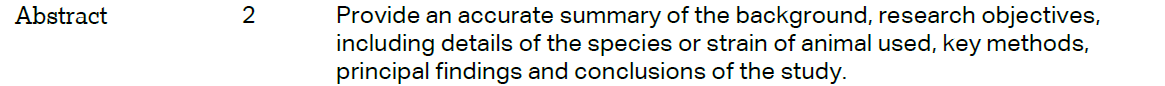 | | | Abstract |  |
| INTRODUCTION | | |  |  |
| 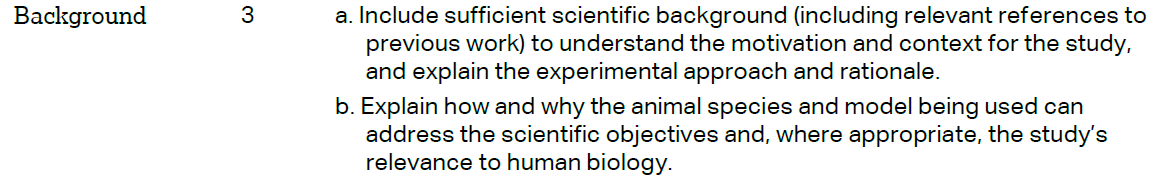 | | | Paragraphs 2-3  Paragraph 2 |  |
| 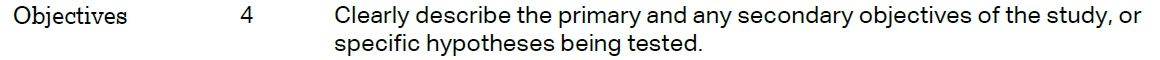 | | | Paragraph 4 |  |
| METHODS | | |  |  |
| 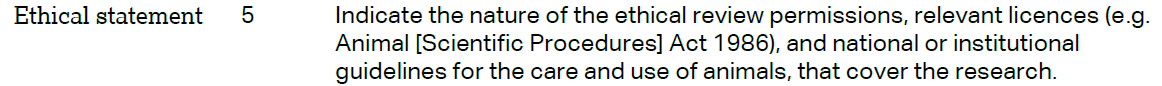 | | | Paragraph 1 |  |
| 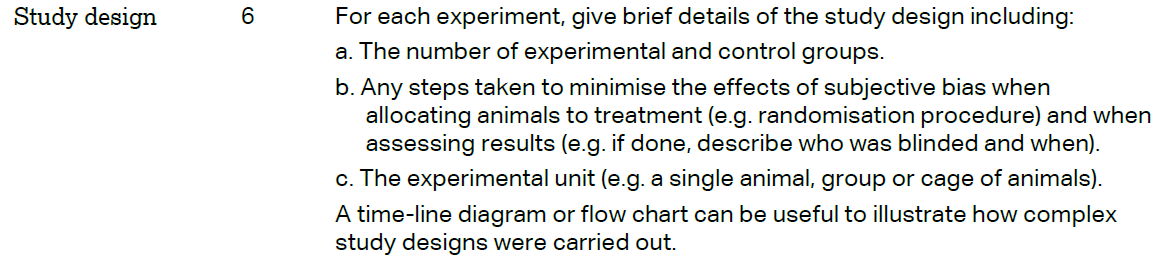 | | | Paragraph 1  −  Paragraphs 2-3, 9-13 |  |
| 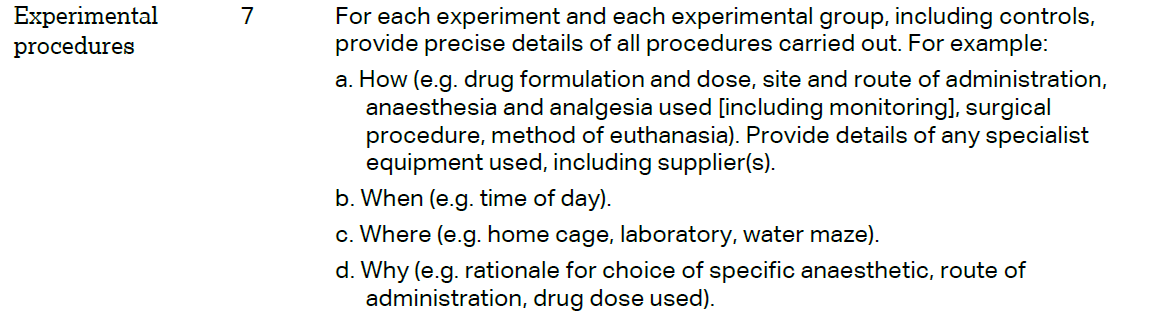 | | | Paragraphs 3-15 |  |
| 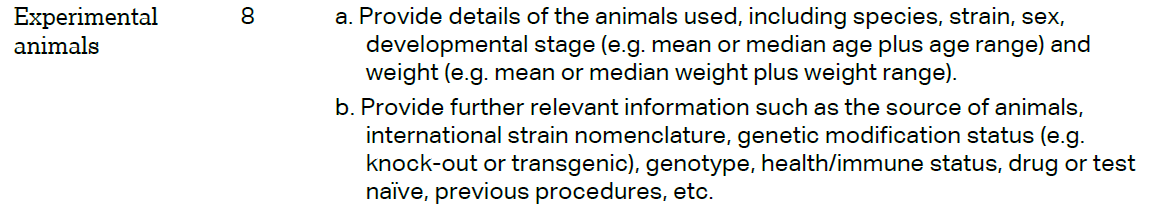 | | | Paragraph 2 |  |

The ARRIVE guidelines. Originally published in *PLoS Biology*, June 2010^1^

| 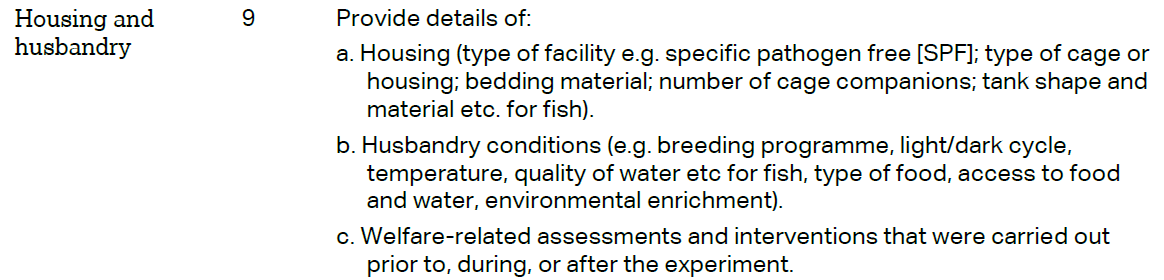 | Paragraphs 2,8 | |
| --- | --- | --- |
| 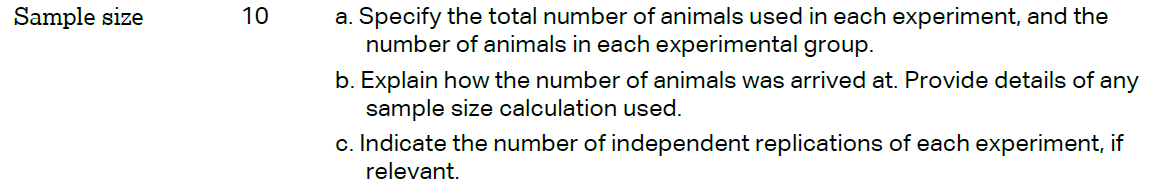 | Paragraphs  2-3, 9-15 | |
| 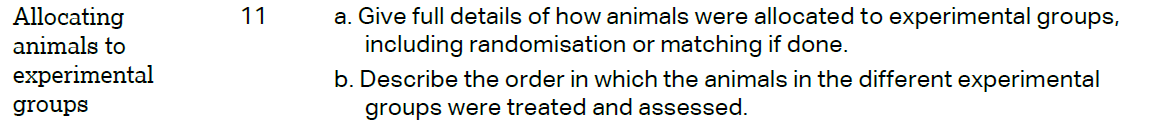 | Paragraphs  9-15 | |
| 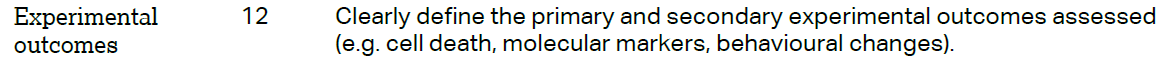 | Paragraphs  9-15 | |
| 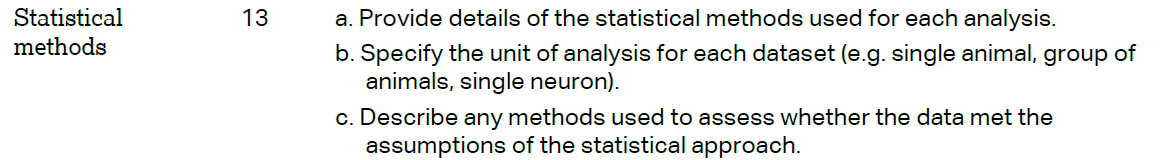 | Paragraph 18 | |
| RESULTS |  | |
| 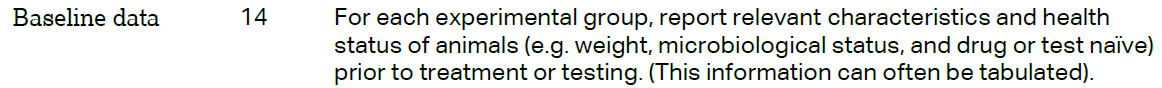 | Methods Paragraph 2 | |
| 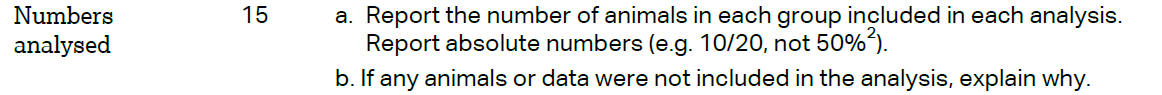 | Methods Paragraphs  9-15 | |
| 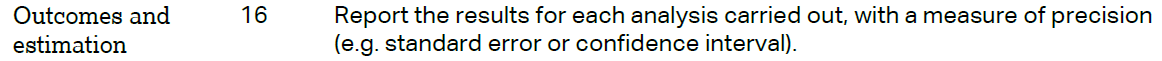 | Paragraphs  1-13 | |
| 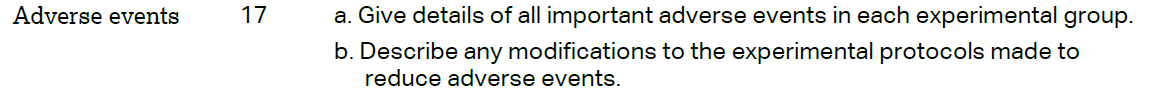 | − | |
| DISCUSSION |  | |
| 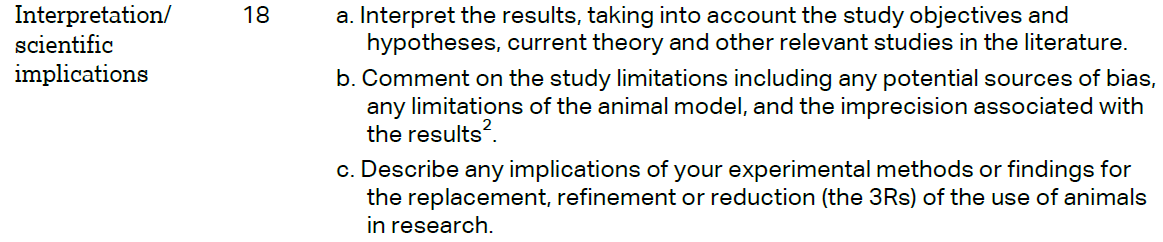 | Throughout  Paragraph 6  − | |
| 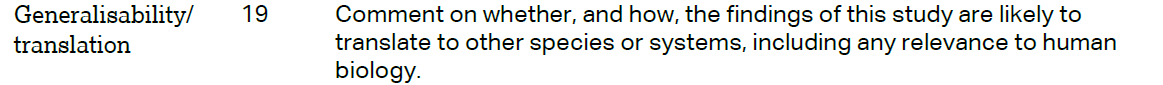 | Paragraph 12 | |
| 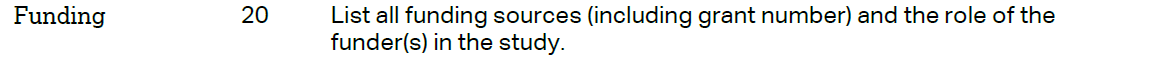 | | Paragraph 13 |


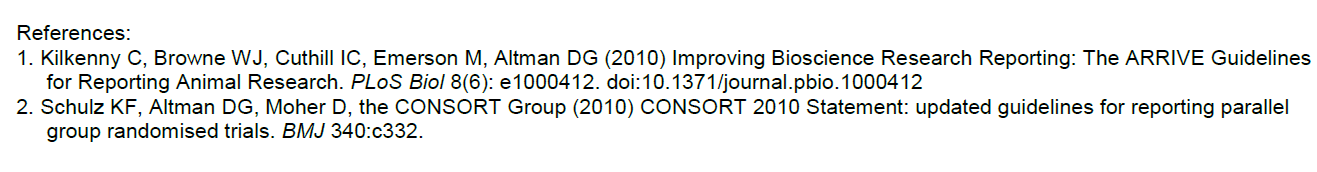

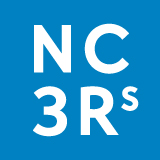

Supplement: S1 File — (DOCX) [file pone.0201519.s001.docx]
